# Does patient-physiotherapist agreement influence the outcome of low back pain? A prospective cohort study

**DOI:** 10.1186/1471-2474-7-76

**Published:** 2006-09-20

**Authors:** Kadija Perreault, Clermont E Dionne

**Affiliations:** 1 Department of Rehabilitation, Faculty of Medicine, Pavillon Ferdinand-Vandry, Laval University, Quebec, Quebec, G1K 7P4, Canada; 2 Centre for Interdisciplinary Research in Rehabilitation and Social Integration, 525, boulevard Wilfrid-Hamel, H-1412, Quebec, Quebec, G1M 2S8, Canada; 3 Population Health Research Unit, Research Centre of the Laval University *Affiliated *Hospital, Hôpital du Saint-Sacrement, 1050 chemin Sainte-Foy Quebec, Quebec, G1S 4L8, Canada

## Abstract

**Background:**

Recent research suggests that agreement between patients' and health professionals' perceptions may influence the outcome of various painful conditions. This issue has received little attention in the context of low back pain and physiotherapy interventions. The current study aimed at exploring the relationship between patient-physiotherapist agreement on baseline low back pain intensity and related functional limitations, and changes in patient outcomes four weeks later.

**Methods:**

Seventy-eight patient-physiotherapist dyads were included in the study. At baseline, patients and physiotherapists completed a Numerical Rating Scale and the Roland-Morris Disability Questionnaire. Patients' perceptions were reassessed over the phone at follow-up.

**Results:**

Using multiple regression, baseline level of patient-physiotherapist agreement on pain intensity was associated with both outcome measures at follow-up. Agreement on functional limitations had no impact on outcomes.

**Conclusion:**

The results of this study indicate that patient-physiotherapist agreement has some impacts on the short-term outcomes of low back pain. Further research is needed to confirm these findings.

## Background

It is well known that low back pain has important negative consequences on the individual and the society. In the last decades, an important body of research has attempted to identify factors associated with the outcome of low back pain. Psychosocial factors have been found to have a predominant influence on the quality of recuperation of individuals with low back pain [1,2]. Past research also highlighted the positive influence of good patient-professional communication on patients' outcomes [3].

According to Prkachin and Craig's Sociocommunications Model of Pain Experience [4,5], a person's experience of pain is expressed to the social world using self-report and/or non-verbal communication. This expression of pain is *decoded *by an observer, such as a healthcare professional, who then reacts to the person's pain [5]. Previous research indicated that agreement between the perceptions of patients and health professionals may be one of the factors affecting the outcome of various painful conditions, such as back problems.

Past studies revealed important discrepancies between the perceptions of patients and healthcare providers on the patient's pain experience [6-9]. Level of agreement has been positively associated with patient outcomes, in terms of reported pain [10-12], overall improvement [13], health status [10,13,14], satisfaction with care [14-17], as well as adherence to treatment and recommendations [18-20]. Implications of low agreement may also include inappropriate assessment of the need to initiate or continue treatment and inadequate appraisal of treatment effectiveness, as suggested by Kwoh [21].

Although most of the evidence tends to indicate that low patient-professional agreement has a negative impact on patient outcomes, only a small number of studies have addressed this issue, and many of these present important methodological limits (e.g. cross-sectional designs). Furthermore, a recent study revealed contrasting results. Indeed, Cremeans-Smith et al. [22] found that physicians' underestimation of patients' pain, rather than agreement, was associated with better patient well-being.

Literature pertaining to patient-healthcare professional agreement is scarce in the area of low back pain. In one of the few published studies, Cedraschi et al. [12] observed that congruence (rather than non-congruence) between patient and therapist (rheumatologist or chiropractor) was associated with the patient's perception of positive evolution of back pain during treatment. The authors determined level of congruence by computing an index of congruence based on patients' and therapists' answers to a 24-item questionnaire covering issues related to back pain, health status and current treatment [12]. In another study, patient-perceived agreement with the physician and the physical therapist regarding the management of low back pain was measured [17]. The authors of this study found that three months after injury, patients who perceived disagreement with their physician catastrophised more about their back pain than those who felt they were in agreement. Patient-perceived agreement with the physical therapist had no impact on outcome [17]. Finally, Staiger et al. [14] measured patient-physician agreement on back pain diagnosis, as well as diagnostic testing and treatment plans. The results of their study indicate that a higher composite agreement score is associated with higher patient satisfaction and health status [14].

To our knowledge, no studies have specifically examined the influence of agreement between patients' and professionals' perceptions of the patient's condition on low back pain outcome, in the context of physiotherapy interventions. Therefore, the objective of this study was to prospectively explore the existence of a relationship between patient-physiotherapist agreement on baseline 1) pain intensity and 2) functional limitations, and changes in patient-reported low back pain intensity and related functional limitations, measured four weeks after baseline. Based on the results of previous research, it was anticipated that higher agreement between patients' and physiotherapists' perceptions would be associated with better improvements in patients' pain intensity and functional limitations.

## Methods

### Design

A prospective longitudinal observational design was used. Measurements were made after the initial physiotherapy consultation (baseline), during which the first assessment was carried out by the physiotherapist, and four weeks later by telephone.

### Settings

Individuals with back pain and their respective physiotherapist were recruited in two private practice physiotherapy clinics of the Quebec City area (Province of Quebec, Canada), between June and October 2003. The Quebec City area is a mainly French-speaking agglomeration of over a million inhabitants that includes more than 70 private physiotherapy clinics.

### Selection of participants

Eligible patients had to: a) be aged 18–75 years, inclusively, b) present non-specific low back pain [23], defined as pain between the 12^th ^rib and the gluteal fold, or cruralgia or sciatica, with or without low back pain [24], c) have had low back pain for twelve weeks or less [25] since its onset or recurrence (acute or sub-acute phase), d) be undertaking treatment at one of the participating clinics, and e) be fluent in French. Patients with and without irradiating pain in the lower limb(s) were included in the study, based on the lack of consensus on the differential evolution of both categories of patients [26]. The exclusion criteria were: a) presence of *Red Flags *[27], which are signs or symptoms of serious spinal pathology or of non-musculoskeletal origin (e.g. cancer, fracture, infection...), b) presence of major co-morbidity, c) pregnancy, d) muteness or deafness, e) being scheduled to undergo major medical or surgical intervention in the four weeks following study entry, and f) legal incompetence.

Eligible physiotherapists had a Bachelor's degree in physiotherapy, were members of the Board of Physiotherapy of the Province of Quebec and practiced in one of the participating clinics. Physiotherapists who had acquired clinical training in addition to physiotherapy, such as osteopathy or acupuncture, were excluded.

### Recruitment procedure

The target sample size for patients was 75, considering an alpha level of 0.05 and a desired statistical power of 0.80, and allowing for an anticipated loss of 20 % of subjects at follow-up and for missing values [28]. This sample size was obtained using a sample size calculation based on an analysis of relationships using Pearson's *r *(bilateral hypothesis) [28]. The final estimated effective statistical power was 88.6 % for pain intensity and functional limitations. This indicates that there was an excellent probability of detecting an *r *≥ 0.35, if this was in fact the real correlation coefficient.

Individuals were recruited by convenience sampling. The research coordinator (KP) carried out this procedure instead of the participating physiotherapists, in order to limit response contamination. For feasibility reasons, the physiotherapists' sample size was limited to the number of eligible and consenting physiotherapists. Written consent was a requirement for patient and physiotherapist participation.

### Main variables and instruments

Patient-physiotherapist agreement constituted the baseline measurements and main independent variables. Agreement was measured for two variables: perceptions of patients' pain intensity and functional limitations. Agreement was operationally defined based on the absolute and signed differences between patients' and physiotherapists' ratings (patient's rating minus physiotherapist's) [29]. Changes in the patients' perceptions of pain intensity and functional limitations four weeks after baseline represented the dependent variables. The same instruments were used to collect both parties' perceptions [29]. The items and instructions of the physiotherapists' questionnaires were modified in order to refer to the patients.

Pain intensity was measured using an 11-point Numerical Rating Scale (NRS) ranging from 0, «no pain», to 10, «worst pain imaginable» [30]. The scale referred to pain felt by the patient on the day of questionnaire completion. The NRS has been found to be valid, reliable and responsive to change [31,32]. It is easy to use [32] and can be administered over the phone [31].

Perceptions of functional limitations were measured using the Roland-Morris Disability Questionnaire (RMDQ), a self-administered back-specific questionnaire derived from the Sickness Impact Profile (SIP) [33]. It contains 24 yes/no items describing difficulties in accomplishing activities because of low back [33] or leg pain [34], on the day of the questionnaire completion. The total score varies from 0, «no disability», to 24, «severe disability». In order to facilitate interpretation, the total score was converted onto a 0–100 scale by dividing it by the number of items answered and multiplying by 100. The RMDQ has been found valid, reliable and sensitive to change [33,35-37]. It is simple to use and can be administered over the phone [38]. A French-Canadian version of this tool was used. It has been employed successfully elsewhere [39].

### Data collection

Immediately after initial consultation, the patients completed: a) a general information questionnaire, b) a questionnaire on pain perceptions, including the NRS, c) the RMDQ, and d) the 14-item Psychological Distress Index (PDI-14) [40,41], a questionnaire adapted from the Psychiatric Symptom Index [42]. At follow-up, the patients' perceptions were reassessed over the phone using the NRS and the RMDQ. Patients then also answered a few questions pertaining to treatment.

After the assessment, the physiotherapists completed the NRS, the RMDQ and responded to questions concerning the consultation. For feasibility reasons, it was tolerated that the physiotherapists completed the questionnaires on the same day of the consultation (rather than immediately after). The physiotherapists were instructed to give their own perceptions of the patients' conditions, rather than what they thought their patients' perceptions were, or what the patients stated during the assessment [43]. The physiotherapists also completed a questionnaire with items on socio-demographic information and their professional practice, at the beginning of the study.

Patients and physiotherapists completed the questionnaires in separate rooms. They were asked not to discuss study participation. The coordinator was present with the patients for the initial data collection, to answer their questions and to verify integrity of the data. The physiotherapists were told to proceed with their usual assessment and treatments. Three physiotherapists and two individuals with back pain who were not involved in the study pre-tested the questionnaires.

### Data analysis

Descriptive statistics were computed to summarize the participants' characteristics and clinical ratings. Results of cross-sectional analyses have been published elsewhere [9]. Absolute and signed difference scores were calculated to measure agreement between the patient's and his/her physiotherapist's ratings for pain intensity and functional limitations. For the signed difference, a positive sign indicated that the patient gave a higher rating than the physiotherapist and a negative sign indicated the opposite. Stepwise multiple regression analyses were conducted to examine the relationships between baseline agreement and changes in patients' perceptions for both variables at follow-up, while controlling for confounders. For thorough exploration of relationships, analyses were conducted using different operational definitions of baseline agreement. Indeed, based on current scientific evidence, there is no consensus regarding the most appropriate way to operationalise this variable. Differences between patients' and physiotherapists' ratings were first entered as continuous absolute variables (easier to interpret than continuous signed differences). Signed difference scores were then modelled as categorical trichotomous variables reflecting patient-physiotherapist agreement, physiotherapist overestimation (physiotherapist's score relative to the patient's) and physiotherapist underestimation (physiotherapist's score relative to the patient's), as proposed by Cremeans-Smith et al. [22]. Patient-physiotherapist agreement was defined as the difference between patients' and physiotherapists' scores, which fell within the range of ± a predetermined threshold. The literature does not offer a clear understanding of what consists of a clinically meaningful difference between a patient's and a professional's score or the accepted difference between scores that represents patient-professional agreement for variables such as pain intensity and functional limitations. Hence, different thresholds were used in the analyses in order to account for different operationalisations of patient-physiotherapist agreement and physiotherapist over- and underestimation. The following thresholds were considered: difference scores within ± 1, 2 and 3 units on the NRS and ± 10, 20 and 30 % on the RMDQ. Changes between baseline and follow-up pain intensity and functional limitations scores (dependent variables) were analysed by entering the patient's rating at follow-up, while controlling for the baseline rating of the same variable. Ratings on the RMDQ, the NRS and the PDI-14 were treated as continuous data in the analyses [28].

Based on the literature, the following potentially confounding variables were considered in the analyses: 1) patient's and physiotherapist's gender and age, as well as patient's level of education, occupation (off work for health-related reason, working full or part-time, not working or retired, student/other), psychological distress, financial compensation, history of low back pain, pain duration and site of pain. In addition, the patient's baseline rating of pain intensity was considered as a possible confounder of the relation between agreement on functional limitations and changes in this variable at follow-up. Patient's baseline rating of functional limitations was also tested for confounding in the relation between agreement on pain intensity and change in pain at follow-up. Confounding was judged at a 20 % threshold of change in the standardised regression coefficient of the independent variable (measure of agreement). Patients' age and gender were forced in the models. In order to verify the stability of the results, a second set of regression analyses was conducted using rank-transformed dependent variables. According to Conover and Iman [44], this procedure is equivalent to conducting non-parametric tests. As suggested by Rothman [45], no adjustments were made for multiple comparisons.

The possibility that patients assessed by the same physiotherapist would be in better agreement and/or more homogeneous at follow-up (cluster effect) was considered. However, calculations of intra-physiotherapist (intra-class) correlation coefficients using multilevel modeling with the GENMOD procedure in SAS indicated that the cluster effect was negligible (data not shown). The number of clusters (physiotherapists) was also considered too small for appropriate application of multilevel analysis [46]. Therefore, analyses were not pursued in this vein.

All the statistical tests were bilateral. Statistical significance was set at α = 0.05. Data were analysed using SPSS version 11.0.

## Results

### Participation rate

Seventy-eight patients and their respective physiotherapist (n = 9) participated in this study, resulting in 78 patient-physiotherapist dyads. The secretaries of the clinics introduced the project to 139 patients. Of the 116 individuals who accepted to be contacted by the research coordinator, 81 were found eligible (69.6 %), but 3 did not participate. It was estimated that 16 patients would have been eligible among the individuals who did not accept to be contacted (n = 23), based on the proportion of patients who were found eligible to participate among the patients who accepted to be contacted by the coordinator (81 eligible/116 accepted to be contacted). Therefore, the overall estimated participation rate for the patients was 61.4 % (number of participating patients/estimated number of eligible patients). Comparative statistics of available data demonstrated that patients excluded and included did not differ in terms of gender and age (data not shown). All physiotherapists were eligible for study participation and none of them refused entry. There were no losses of participants to follow-up (100 % retention rate), which was carried out a mean of 27.8 ± 1.8 days after baseline (range 25.0 – 35.0).

### Characteristics of participants

Selected characteristics of participating patients and physiotherapists are presented in Table 1. Thirty-eight men and 40 women with back pain aged between 24 and 73 years were included in the study. A majority of individuals were highly educated (52.6 % attended university) and married (or living as married) (79.5 %). Only a minority of subjects was off work for health-related reasons (10.3 %) or receiving a financial compensation for back pain (16.7 %). The mean psychological distress score was low (PDI-14 = 12.5/100). At follow-up, 38.5 % of participating patients were still receiving physiotherapy treatments. The mean number of physiotherapy sessions received in four weeks was 7.5 ± 1.7 (range 1.0 – 16.0, including first visit).

The physiotherapists were full-time workers and their highest level of education was a Bachelor's degree. Continuing education in which they had participated in the past included McKenzie Technique, Manual Therapy and Sports Physiotherapy. The physiotherapists' general treatment modalities included education, manual therapy, various exercises, mechanical traction, electrotherapy and thermal modalities.

### Relationships between agreement and changes in outcome

Mean ratings and absolute differences between patients' and physiotherapists' ratings are provided in Table 2. Using the rank-transformed dependent variables in the analyses lead to the same conclusions than with the untransformed variables in all models, except for the relationship between patient-physiotherapist agreement on pain intensity and changes in functional limitations at follow-up. Since using the rank-transformation limits the interpretation of data, the results using the untransformed dependent variables are presented, except for the above-mentioned relationship for which the model is based on rank-transformation.

Tables 3 and 4 present the results of statistical modeling using a threshold of ± 3 units on the NRS and 30 % on the RMDQ. Number of dyads per group corresponding to physiotherapist underestimation and overestimation are provided in the tables. Hence, the rest of the n = 78 patient-physiotherapist dyads were considered to be in agreement. When modeled as absolute continuous difference scores or trichotomous variables with physiotherapist over- or underestimation by 1 or 2 units on the NRS and by 10 or 20 % on the RMDQ, agreement at baseline was not significantly associated with changes in pain intensity or functional limitations at follow-up (*p *> 0.05) (data not shown to prevent from unduly prolonging this article). Data from all dyads are presented since exclusion of the two most extreme cases did not affect the results.

Dyadic agreement on pain intensity, but not functional limitations, was found to be associated with changes in pain intensity after four weeks. Patients who rated pain intensity more than 3 units higher than their physiotherapist on the NRS reported reduced pain at follow-up compared to baseline. This was the case in four dyads. Patients in these four dyads had pain duration at baseline between 2 and 10 days and were not receiving compensation. They completed follow-up questionnaires 27 or 28 days after baseline. They had not changed their medication since baseline, had not consulted another professional (except a referring doctor in one case), and only one of them was still undergoing treatment.

Agreement on pain intensity also had an impact on 4-week changes in functional limitations. Patients who rated pain intensity more than 3 units lower than their physiotherapists on the NRS presented reduced functional limitations at follow-up compared to baseline. This was the case for only two dyads, in which patients had had pain for 1 and 7 days, were not receiving financial compensation, had not consulted another professional and were no longer receiving treatments at follow-up (carried out 27 and 33 days after baseline). Both patients reported no pain and functional limitations four weeks after baseline. There was no significant association between level of agreement on functional limitations and patients' changes for this variable at follow-up.

## Discussion

The aim of this study was to explore the existence of a relationship between patient-physiotherapist agreement and short-term patient outcomes that are most relevant in low back pain. Dyadic agreement on perceptions of pain intensity was associated with changes in patient outcomes, in the short term. Lower patient-physiotherapist agreement on pain intensity was associated with positive changes in pain intensity and functional limitations at follow-up compared to baseline. Agreement on perceptions of functional limitations had no impact on pain intensity and functional limitations after four weeks.

The finding that higher disagreement on pain intensity was favourable to pain outcome was surprising. Indeed, most other studies demonstrated that higher discrepancies between patient and professional are detrimental to patient care and outcome [10-13,15,16,18-20,47], or have no effects [48]. To our knowledge, Cremeans-Smith et al. [22] are the only other authors to have found similar results to ours. They examined the influence of agreement between patients with osteoarthritis and rheumatologists about perceptions of pain severity on psychological well-being in a cross-sectional study [22]. They found that physician underestimation of patients' pain severity was associated with better patient self-efficacy and positive affect. It is possible that when physiotherapists underestimate pain compared to their patients, their management approach portrays to the patients a less dramatic picture of their condition, therefore offering them reassurance. Cremeans-Smith et al. [22] similarly suggested that physician underestimation of pain may portray to the patients an optimistic view of intervention. These hypotheses however contradict Bass et al.'s [10] suggestion that good patient-provider agreement (in this case, on the nature of the problem) might provide healing reassurance to the patient. Another possible explanation for our finding may be that by rating pain lower than their patients, physiotherapists encourage them to play an active role in the intervention, rather than be passive recipients of treatment. Active participation of the patient has been recommended as a means of improving the outcome of low back pain [49].

It was furthermore surprising to find that physiotherapist's overestimation of pain was associated with lower functional limitations at follow-up. No reports of the specific effect of a professional's overestimation of pain have been presented in the literature to our knowledge. One may suppose that the physiotherapist's overestimation of pain intensity may have led him or her to pay more attention to the patient's pain. This may be perceived positively by the patients and therefore may favour the quality of the patient-physiotherapist relationship and consequently improve outcome. Still, positive clinical impact of physiotherapist overestimation of pain is hard to explain at this point. It is possible that this finding reflects a statistical effect, such as regression to the mean. In addition, since this result was obtained using the rank-transformed dependent variable, it is not possible to interpret the size of the effect, which may not represent a clinically important change.

Our findings suggest that high patient-physiotherapist discrepancies (more than a 3-unit difference in ratings on the NRS) are needed to have a clinical impact in the short term. Observations of the regression coefficients in the relationships between agreement on pain intensity and changes in both outcome measures suggest that high differences between patients' and physiotherapists' ratings may be associated with reduced pain and functional limitations at follow-up compared to baseline, although only two associations were found to be statistically significant. These results should be considered with extreme caution. Indeed, very few dyads were involved in these statistically significant associations. All the patients in these dyads were in the acute phase of pain and had an excellent outcome of their back pain problem after four weeks. Furthermore, the size of the effect was generally small in most models, as indicated by the values of the regression coefficients and adjusted *R*^2 ^(Tables 3 and 4), which suggests that the influence of agreement on the measured outcomes may not be clinically important.

This study has several limitations. The sample size was relatively small. This may have yielded higher variability in the agreement measures (high difference scores were recorded scarcely) and therefore masked real effects of agreement. Because of the non-random sampling method of recruitment and the particular socio-demographic characteristics of the participating patients (highly educated, full-time workers...), the results of this study may only be generalisable to clinical encounters between patients and physiotherapists with characteristics similar to the study participants'. Measures of agreement may have been influenced by the fact that patients and physiotherapists were informed of the aims of the study before entry. Knowledge of this information may have affected the way both parties answered the questionnaires [50]. Furthermore, physiotherapists were not blind to patient participation. They may have changed their interventions with participating patients, although they were instructed not to do so. The natural history of low back pain and differences in physiotherapy treatments (e.g. number of sessions) and other interventions (e.g. medication) between patients may have also had varying effects on patient outcomes. Finally, analytical issues such as missing important confounding variables may have negatively affected our results [51].

## Conclusion

Up to now, few studies have evaluated the impact of patient-provider agreement on patient outcomes, especially in a prospective manner. Studying the impact of patient-physiotherapist agreement on the outcome of low back pain represents an innovative and relevant project as highlighted by the findings of recent research on low back pain. Still, the results of the current study do not allow us to formulate any definite conclusions about the influence of patient-physiotherapist agreement on changes in patient outcomes. Replication of our results is necessary before any recommendations can be made to physiotherapists. Further research is also warranted to verify the impact of patient-physiotherapist agreement on other outcome variables, in the longer term, and in different populations.

## Competing interests

The author(s) declare that they have no competing interests.

## Authors' contributions

KP contributed to the conception and design of the research, acquired the data and participated in analysing and interpreting the data, as well as in writing and reviewing the article.

CED contributed to the conception and design of the research and participated in analysing and interpreting the data, as well as in writing and reviewing the article

Both authors read and approved the final manuscript.

## Pre-publication history

The pre-publication history for this paper can be accessed here:



## Figures and Tables

**Table 1:** Selected characteristics of patients and physiotherapists

**Variable**	**n (%) **	**or**	**mean (SD)**
**1) Patients (n = 78)**			
Gender			
Men	38 (48.7)		
Women	40 (51.3)		
Age (years; range: 24.2 – 73.0)			47.5 (10.6)
Marital status			
Never married	12 (15.4)		
Married or living as married	62 (79.5)		
Divorced/Separated/Widowed	4 (5.1)		
Current occupation			
Off work for health reasons	8 (10.3)		
Full/Part-time work	50 (64.1)		
Does not work/Retired	17 (21.8)		
Student/Other	3 (3.8)		
Highest level of education (part or complete)			
Primary school	1 (1.3)		
Junior/High school	15 (19.2)		
Junior College	21 (26.9)		
University	41 (52.6)		
Current financial compensation			
Yes	13 (16.7)		
No	65 (83.3)		
Previous low back pain			
Yes	63 (80.8)		
No	15 (19.2)		
Previous physiotherapy treatment for low back pain			
Yes	37 (47.4)		
No	41 (52.6)		
Previous treatment at participating clinic			
Yes	43 (55.1)		
No	35 (44.9)		
Previous treatment with same physiotherapist			
Yes	13 (16.7)		
No	65 (83.3)		
Physiotherapist was referred to patient by a third party			
Yes	22 (28.2)		
No	56 (71.8)		
Pain duration (days; range: 1.0 – 90.0)			22.0 (23.7)
Phase of pain			
Acute (0 – 4 weeks)	59 (75.6)		
Sub-acute (4 – 12 weeks)	19 (24.4)		
Pain location			
Back only/Back and buttocks	46 (59.0)		
Back and leg, above the knee	8 (10.3)		
Back and leg, below the knee	15 (19.2)		
Leg or buttocks only	9 (11.5)		
Psychological distress (%; range: 0.0 – 50.0)			12.5 (10.5)
**2) Physiotherapists (n = 9)**			
Gender			
Men	4 (44.4)		
Women	5 (55.6)		
Age (years; range: 23.2 – 50.1)			33.2 (9.4)
Marital status			
Never married	4 (44.4)		
Married or living as married	5 (55.6)		
Personal experience of low back pain			
Yes	8 (88.9)		
No	1 (11.1)		
Professional experience (years; range: 0.0 – 22.2)			8.6 (7.6)
Proportion of patients with low back pain in daily practice (%; range: 30.0 – 50.0)			38.9 (8.2)

**Table 2:** Mean ratings^a ^for both outcome measures at baseline and follow-up

	**Baseline**			**Follow-up**
	
	**Patients**	**Physiotherapists**	**Absolute difference**	**Patients**
Pain intensity^b^	4.5 (2.3)	3.9 (2.0)	1.5 (1.4)	2.0 (2.3)
Functional limitations^c^	40.4 (23.0)	27.5 (22.4)	17.7 (15.0)	16.2 (19.8)

^a^Mean (SD)

^b^Range: 0.0 – 10.0

^c^Range: 0.0 – 100.0

**Table 3:** Relationship between agreement at baseline and change in pain intensity at follow-up

**Agreement on:**	**β**	**95 % CI**^**c**^	***P***
**1) Pain intensity**			
Intercept	-.248	-2.699 – 2.203	.84
Baseline pain intensity	.458	.225 – .692	<.01*
Physiotherapist^a^:			
Underestimation (n = 4)	-3.328	-5.733 – -.922	<.01*
Overestimation (n = 2)	-.976	-3.998 – 2.046	.52
			
**2) Functional limitations**			
Intercept	-.829	-4.038 – 2.380	.61
Baseline pain intensity	.235	.038 – .433	.02*
Physiotherapist^b^:			
Underestimation (n = 13)	.301	-.913 – 1.516	.62
Overestimation (n = 1)	-1.979	-5.963 – 2.005	.33

*p < .05

^a^Physiotherapist over- or underestimation by > 3/10 on the NRS, with control for patient's age, gender and pain duration

^b^Physiotherapist over- or underestimation by > 30 % on the RMDQ, with control for patient's age, gender, current financial compensation and pain duration, as well as physiotherapist's age

^c^CI = Confidence interval

Total model (pain intensity) adjusted *R*^2 ^= .227, *p *< .01

Total model (functional limitations) adjusted *R*^2 ^= .331, *p *< .01

**Table 4:** Relationship between agreement at baseline and change in functional limitations at follow-up

**Agreement on:**	**β**	**95 % CI**^c^	***P***
**1) Pain intensity**^a^			
Intercept	8.436	-14.520 – 31.392	.47
Baseline functional limitations	.334	.125 – .543	< .01*
Physiotherapist^b^:			
Underestimation (n = 4)	-12.618	-32.783 – 7.546	.22
Overestimation (n = 2)	-28.738	-55.963 – -1.513	.04*
			
**2) Functional limitations**			
Intercept	-10.547	-33.707 – 12.613	.37
Baseline functional limitations	.173	-.058 – .403	.14
Physiotherapist^c^:			
Underestimation (n = 13)	5.938	-4.362 – 16.238	.25
Overestimation (n = 1)	21.142	-9.917 – 52.202	.18

*p < .05

^a^Model using rank-transformed dependent variable

^b^Physiotherapist over- or underestimation by > 3/10 on the NRS, with control for patient's age, gender and psychological distress

^c^Physiotherapist over- or underestimation by > 30 % on the RMDQ, with control for patient's age, gender, baseline pain intensity, psychological distress, pain duration and site of pain

^d^CI = Confidence interval

Total model (pain intensity) adjusted *R*^2 ^= .288, *p *< .01

Total model (functional limitations) adjusted *R*^2 ^= .456, *p *< .01
